# Renal pathological analysis using galactose-deficient IgA1-specific monoclonal antibody is a strong tool for differentiation of primary IgA nephropathy from secondary IgA nephropathy

**DOI:** 10.1007/s13730-020-00508-3

**Published:** 2020-07-16

**Authors:** Mingfeng Lee, Hitoshi Suzuki, Rina Kato, Yusuke Fukao, Maiko Nakayama, Toshiki Kano, Yuko Makita, Yusuke Suzuki

**Affiliations:** Department of Nephrology, Juntendo University Faculty of Medicine, 2-1-1 Hongo, Bunkyo-ku, Tokyo, 113-8421 Japan

**Keywords:** IgA nephropathy, Galactose-deficient IgA1, KM55, IgA deposition

## Abstract

In several cases with IgA nephropathy (IgAN), differential diagnosis is difficult due to the complication with other systemic diseases which can induce secondary IgAN. Recently, we demonstrated that immunostaining with galactose-deficient IgA1-specific monoclonal antibody (KM55 mAb) specifically showed positive in primary IgAN cases. Here, we report four cases which we could make definitive diagnosis by immunohistological analysis using KM55 mAb. The underlying systemic diseases are rheumatoid arthritis (RA), systemic lupus erythematosus (SLE), hepatitis C (HCV) and Crohn’s disease (CD). Renal pathological findings in the four cases revealed mesangial proliferative glomerulonephritis with IgA and C3 deposits. Immunostaining with KM55 mAb was positive for three cases complicated with RA, SLE and CD, respectively. Thus, these three cases were diagnosed as primary IgAN and treated with tonsillectomy and steroid pulse therapy. These three cases finally achieved clinical remission. On the other hand, the case with HCV showed negative for KM55. Finally, we diagnosed as HCV-related nephropathy and successfully treated by antiviral agents. These cases suggested KM55 mAb is a strong tool to differentiate primary IgAN from secondary IgAN.

## Introduction

IgA nephropathy (IgAN) is the most common primary glomerulonephritis worldwide. It is defined as primary glomerulonephritis with predominant glomerular IgA deposition. However, glomerular IgA deposition is found not only in IgAN but also in other systemic diseases like gastrointestinal and liver diseases, autoimmune disorders, neoplasia and infections [1]. It is important to differentiate primary IgAN from secondary IgAN, because therapeutic strategy is different depending on the underlying primary disease. However, it is not easy to distinguishing primary IgAN from secondary IgAN, if the patient has comorbidities which could induce secondary IgAN. Previous studies have demonstrated that galactose-deficient IgA1 (Gd-IgA1) is specifically involved in the pathogenesis of primary IgAN [2, 3]. Recently, the method of measuring serum levels of Gd-IgA1 using monoclonal antibody against Gd-IgA1 (KM55 mAb) has been established [4]. Besides, we reported that KM55 mAb could detect Gd-IgA1 deposition in glomeruli in the cases of primary IgAN, but not in other renal diseases [5]. Thus, histological analysis using KM55 mAb may be a strong tool for differentiating primary IgAN from secondary IgAN. Here, we show 4 cases which we could make definitive diagnosis by immunostaining with KM55 mAb.

## Case reports

### Case 1

A 44-year-old woman had presented hematuria and proteinuria from 7 years ago. She was admitted to perform renal biopsy in our hospital. Besides, she has showed PIP joints pain for several years. Both rheumatoid factor (RF) and anti-cyclic citrullinated peptide antibody (anti-CCP) showed positive. Thus, rheumatoid arthritis (RA) was diagnosed by rheumatologist.

Blood examination showed Cr 0.43 mg/dl, eGFR 121.8 mL/min/1.73 m^2^, IgG 1429 mg/dl, IgA 653 mg/dl, C3 72 mg/dl, RF 734.4 IU/ml, anti-CCP-Ab 6.2 IU/ml. Antinuclear antibodies (ANA), myeloperoxidase anti-neutrophil cytoplasmic antibodies (MPO-ANCA), proteinase3 anti-neutrophil cytoplasmic antibodies (PR3-ANCA), anti-glomerular basement membrane antibodies (anti-GBM), and cryoglobulins were negative. Urinalysis showed urine protein–creatinine ratios (UPCR) 2.4 g/gCr, urinary red blood cells (U-RBC) 11–15/high-power field (HPF).

**Fig. 1:**
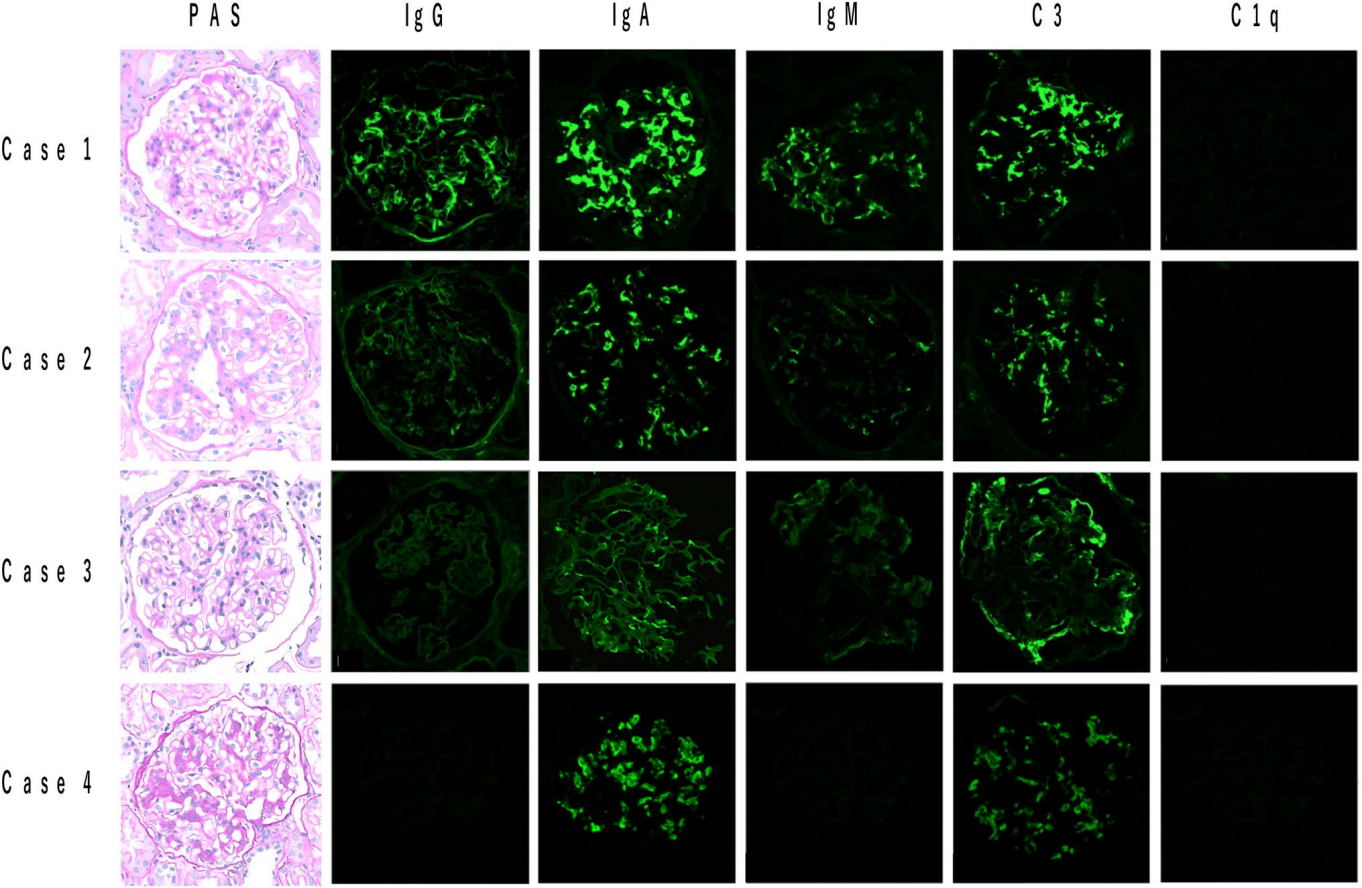
Light microscopic findings and immunofluorescence staining of IgG, IgA, IgM, C3 and C1q in cases 1–4. All four cases showed mesangial proliferative lesions and positive for IgA and C3. *PAS* Periodic Acid–Schiff stain. Original magnification ×400

Light microscopic finding (LM) revealed mesangial cell proliferation and increase of mesangial matrix as well as endocapillary proliferation. Immunofluorescence analysis (IF) revealed mesangial deposition of IgA and C3. Mesangial and capillary deposition of IgG and IgM also noted. (Fig. 1).

**Fig. 2:**
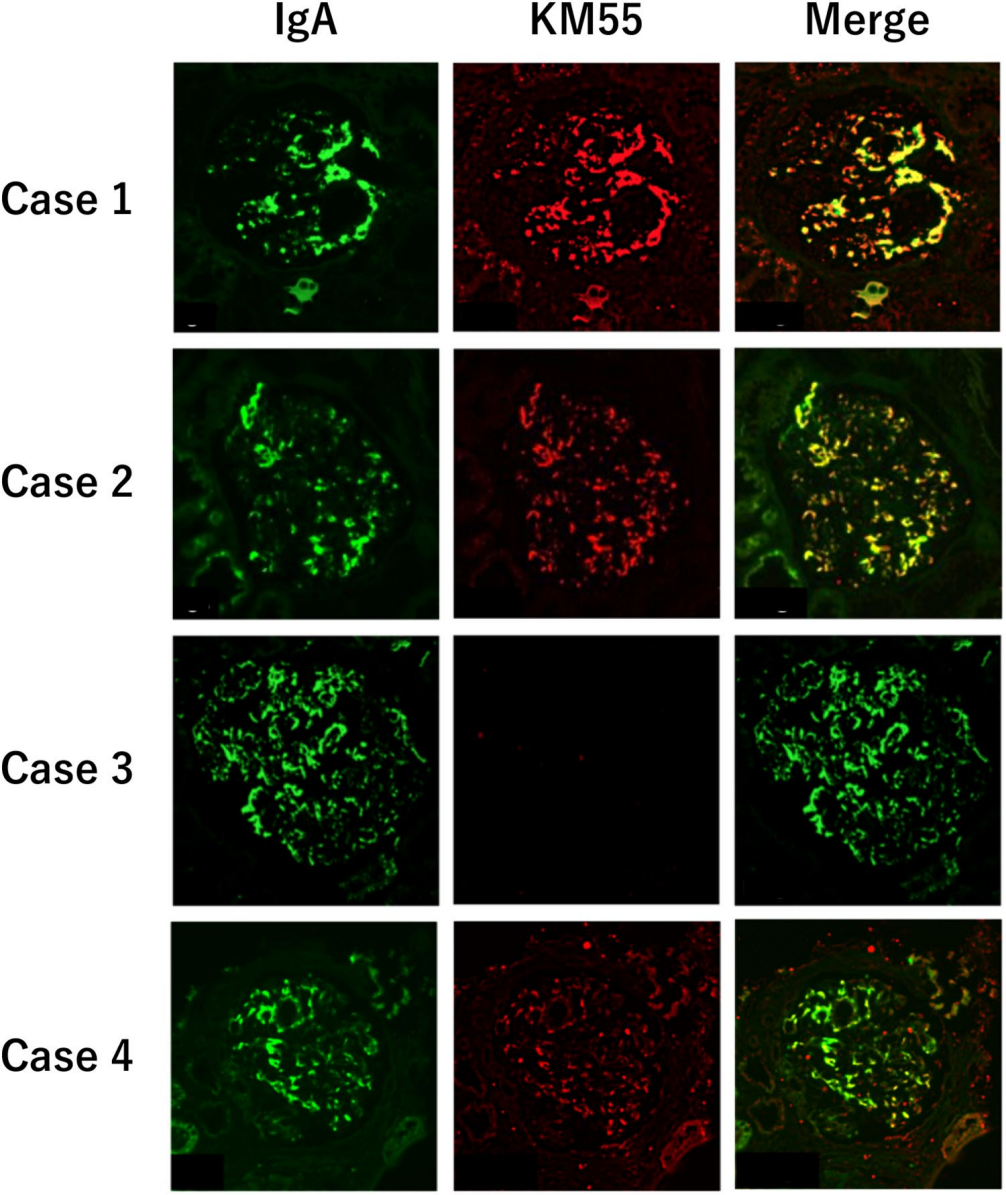
Immunofluorescence staining of IgA, KM55 and merge image in cases 1–4. Cases 1, 2 and 4 showed positive for KM55. Case 3 showed negative for KM55. Original magnification ×400

Based on the pathological findings, we diagnosed as IgAN. To exclude secondary IgAN caused by RA, immunostaining with KM55 mAb was performed. Finally, we diagnosed as primary IgAN due to positive staining of KM55 mAb colocalized with IgA deposit area (Fig. 2).

**Fig. 3:**
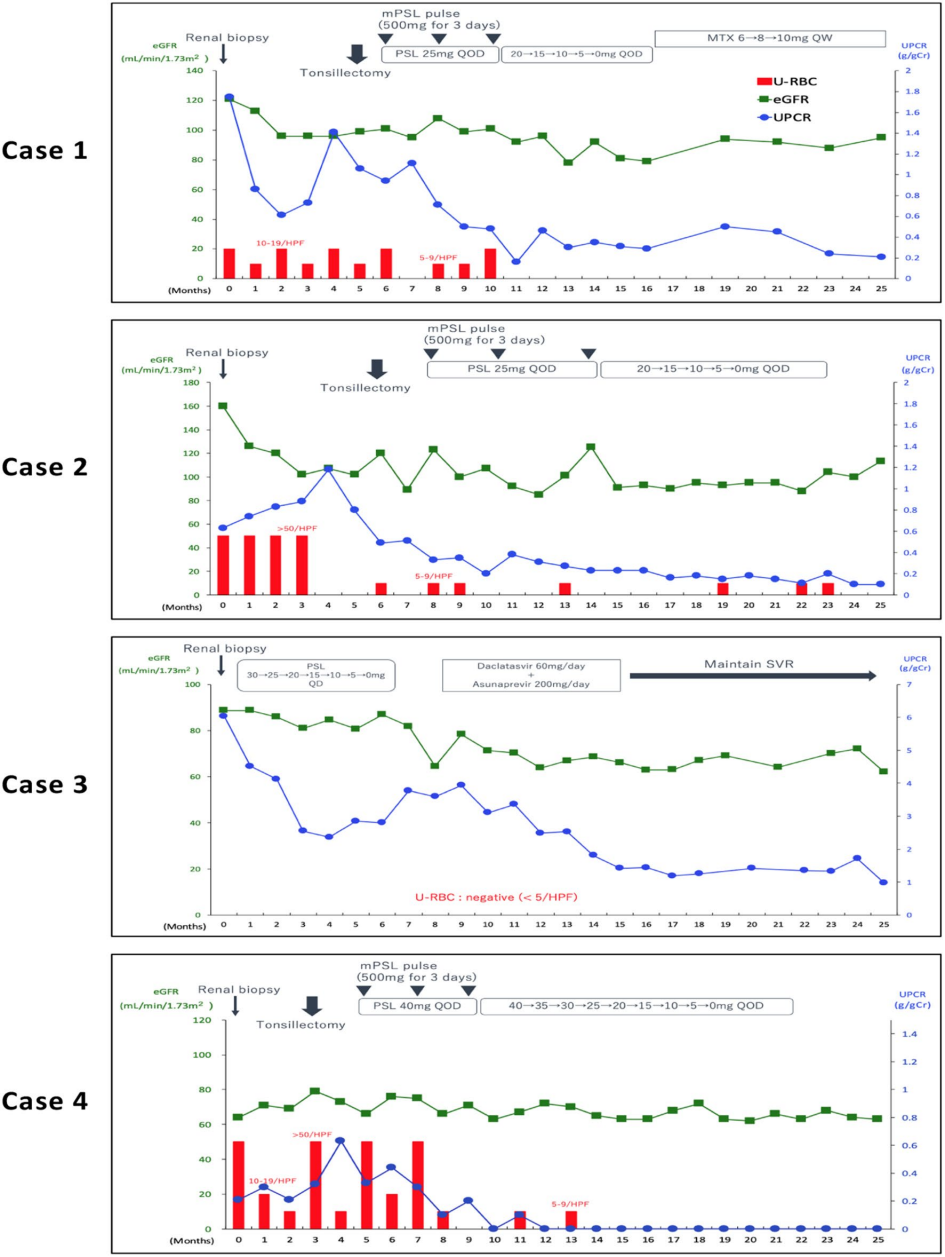
Clinical courses of cases 1–4. *mPSL* methylprednisolone, *PSL* prednisolone, *MTX* methotrexate, *QD* once daily, *QOD* every other day, *QW* once a week, *eGFR* estimated glomerular filtration rate [mL/min/1.73 m^2^], *UPCR* urine protein–creatinine ratio [g/gCr], *U-RBC* urinary red blood cells, *HPF* high-power field, *SVR* sustained virological response

According to the diagnosis of primary IgAN, tonsillectomy with steroid pulse (TSP) therapy was performed. Urinary abnormalities has been gradually improved and achieved clinical remission (CR) afterward (Fig. 3). CR defined as three consecutive negative results over a 6-month period in urinary occult blood tests; urinary sediment red blood cell count of < 5/HPF; and urinary protein of < 0.3 g/day [6]. After TSP therapy, serum levels of IgA and Gd-IgA1 significantly decreased (Table 1).

**Table 1:** Serum levels of IgA, galactose-deficient IgA1 (Gd-IgA1), and Gd-IgA1/IgA ratio before and after tonsillectomy with steroid pulse (TSP) therapy (in cases 1, 2 and 4) and serum levels of IgA, Gd-IgA1 and Gd-IgA1/IgA ratio at diagnosis (in case 3)

	IgA (mg/dl)	Gd-IgA1 (ng/ml)	Gd-IgA1/IgA
Case 1 (Before TSP)	653	8542.2	13.1
Case 1 (After TSP)	388	4746.8	12.2
Case 2 (Before TSP)	282	7277.0	25.8
Case 2 (After TSP)	258	4911.5	19.0
Case 3 (At diagnosis)	338	3164.2	9.4
Case 4 (Before TSP)	502	8570.6	17.1
Case 4 (After TSP)	323	5063.3	15.7

Clinical symptoms of RA initially getting better by steroid therapy. However, after tapering steroid dosage, PIP joints pain has gradually getting worse without relapse of urinary abnormalities. Thus, treatment with methotrexate (MTX) was started after finishing steroid therapy. Currently, the disease activity of RA has been under control with MTX treatment and urinary findings have also maintained remission.

### Case 2

A 42-year-old woman who was diagnosed as systemic lupus erythematosus (SLE) when she was 39 years old with the findings of polyarthritis, positive for ANA (1:640, homogeneous and speckled pattern) and anti-ds-DNA Ab, lymphocytopenia, and proteinuria (0.5–1.0 g/gCr). She had been followed by rheumatologist without any treatment. However, proteinuria and hematuria had been persisted. Thus, a renal biopsy was performed for definitive diagnosis.

Blood examination showed Cr 0.35 mg/dl, eGFR 177.8 mL/min/1.73m^2^, IgG 1214 mg/dl, IgA 282 mg/dl, C3 103 mg/dl. ANA, anti-ds-DNA. anti-SS-A, anti-SS-B, RF, and anti-CCP were positive. MPO-ANCA, PR3-ANCA, anti-GBM, and cryoglobulins were negative. Urinalysis showed UPCR 0.64 g/gCr and U-RBC > 50/HPF.

LM revealed mild mesangial cell proliferation and increase of mesangial matrix. IF showed mesangial deposition of IgA and C3, but negative for C1q that is atypical for lupus nephritis (Fig. 1). Based on the pathological findings, we could not diagnose definitively IgAN or lupus nephritis. Then, immunostaining with KM55 mAb was performed and showed its positive staining colocalized with IgA (Fig. 2). Finally, we diagnosed as primary IgAN. After TSP therapy, urinary abnormalities have been gradually improved and achieved CR (Fig. 3). Moreover, level of Gd-IgA1 significantly decreased (Table 1).

Disease activity of SLE had been relatively stable during steroid usage. However, after discontinuation of steroid treatment, polyarthritis exacerbated and serum level of anti-ds-DNA Ab gradually elevated. She has been followed by rheumatologist. Currently, induction of additional immunosuppression therapy is considered. Both proteinuria and hematuria have been able to maintain remission.

### Case 3

A 63-year-old man had presented with proteinuria (0.5–1.0 g/gCr) from 7 years ago. Proteinuria progressed to around 5.0 g/gCr. A renal biopsy was performed for definitive diagnosis. Besides, he had hepatitis C (HCV) without any anti-viral treatment.

Blood examination showed Cr 0.74 mg/dl, eGFR 114.3 mL/min/1.73m^2^, IgG 1905 mg/dl, IgA 338 mg/dl, C3 120 mg/dl. ANA, RF, MPO-ANCA, PR3-ANCA, anti-GBM and cryoglobulins were negative. HCV-RNA showed as 5.6 LogIU/mL. HCV subtyping showed type 1b. Urinalysis showed UPCR 3.6 g/gCr and U-RBC showed 1–4/HPF. Serum level of Gd-IgA1 was low, compared with cases 1, 2 and 4 (Table 1).

LM revealed mesangial cell proliferation and increase of mesangial matrix as well as endocapillary proliferation. IF showed slightly positive for IgA and C3 in mesangial area (Fig. 1). IgG also showed weakly positive in capillary area. For the presence of active inflammatory lesions, such as endocapillary proliferation, we diagnosed as active IgAN with massive proteinuria.

Initially, we considered to arrange steroid pulse therapy to treat acute lesions. However, for the complication of HCV, steroid therapy would be the risk of its activation. We decided to administer oral steroid therapy instead of steroid pulse therapy after discussing with gastroenterologist.

After informed consent, we started steroid therapy with close monitoring of liver function. However, improvement of proteinuria was limited and the liver function gradually getting worse. Thus, we stopped steroid therapy and performed immunostaining with KM55 mAb for definitive diagnosis. Finally, we diagnosed as HCV-related nephropathy (HCV-RN) due to negative staining of KM55 mAb in glomeruli (Fig. 2).

After the diagnosis of HCV-RN, we consulted gastroenterologist for HCV treatment. Antiviral therapy with daclatasvir and asunaprevir was arranged. Proteinuria has been gradually improved by the antiviral treatment (Fig. 3). HCV also achieved sustained virological response (SVR).

### Case 4

A 35-year-old man presented with proteinuria and hematuria which were noted by regular health check. No abnormal urinalysis was noted until three years ago. A renal biopsy was performed for definitive diagnosis. He was diagnosed with Crohn’s disease (CD) when he was 29 years old. The disease activity of CD was stable under treatment with mesalazine and adalimumab.

Blood examination showed Cr 1.06 mg/dl, eGFR 76.6 mL/min/1.73 m^2^, IgG 1304 mg/dl, IgA 502 mg/dl, C3 93 mg/dl, CRP 0 mg/dl. ANA, RF, MPO-ANCA, PR3-ANCA, anti-GBM and cryoglobulins were negative. Urinalysis showed UPCR 0.56 g/gCr and U-RBC > 50/HPF.

LM revealed mesangial cell proliferation and increase of mesangial matrix. IF showed positive for IgA and C3 in mesangial area (Fig. 1). To distinguish primary IgAN and secondary IgAN with CD, immunostaining with KM55 mAb was performed and showed its positive staining colocalized with IgA (Fig. 2). Finally, we diagnosed as primary IgAN. After TSP therapy, urinary abnormalities have been gradually improved and achieved CR (Fig. 3). Furthermore, serum levels of IgA and Gd-IgA1 significantly decreased (Table 1).

## Discussion

There are increasing evidences that Gd-IgA1 play a pivotal role in the pathogenesis of IgAN [7]. Elevations of serum Gd-IgA1 levels and mesangial deposition of Gd-IgA1 were reported in patients with IgAN [3, 5]. Multi-hit hypothesis which includes (Hit 1) production of Gd-IgA1, (Hit 2) IgG or IgA autoantibodies that recognize Gd-IgA1, (Hit 3) their subsequent immune complexes formation and (Hit 4) glomerular deposition was advocated as the most probable pathogenesis of IgAN [2].

Gd-IgA1 is thought to be induced by abnormal mucosal immune responses mainly at the upper respiratory tract including tonsil [8]. In fact, clinical efficacy of tonsillectomy in patients with IgAN has been reported by meta-analysis [9] and recent large retrospective cohort study with propensity score matching [10]. Besides, our group also reported decrease of serum level of Gd-IgA1 just after tonsillectomy associated with the improvement of hematuria [11].

Secondary IgAN is thought to be containing a wide disease spectrum [1]. In the clinical settings, secondary IgAN should be taken into account when systemic comorbidities exist. However, differential diagnosis of primary IgAN from secondary IgAN is not easy, because there are no specific histological features to distinguish them. Secondary IgAN can be diagnosed only by the effectiveness of treating underlying systemic comorbidities. At least, tonsillectomy do not improve secondary IgAN. Whether secondary IgAN shares the common pathogenic process with primary IgAN or not is still unclear and needs further investigation.

According to the previous report, glomerular Gd-IgA1 was specifically detected in IgAN but not in the other types of glomerular diseases [5]. Thus, if Gd-IgA1 involvement is proved by immunohistochemical analysis with KM55 mAb, abnormal mucosal immune response might be related to the pathogenic process, and tonsillectomy might be useful for disease control.

In the present cases, the case with RA, SLE and CD (cases 1, 2, and 4) showed positive for KM55 mAb and no correlation between the disease activity of systemic disease and urinary abnormalities. Thus, we diagnosed as primary IgAN. Those three cases showed improvement of urinary abnormalities by TSP therapy. To demonstrate that TSP therapy improved mesangial inflammation caused by Gd-IgA1 deposition diagnosed by KM55 immunostaining, we measured the level of Gd-IgA1 in all cases by KM55 enzyme-linked immunosorbent assay (ELISA) and showed in Table 1. It is clearly indicated that serum levels of IgA, Gd-IgA1 and Gd-IgA1/IgA ratio decreased by TSP therapy. On the other hand, the case with HCV (case 3) showed negative for KM55 mAb. Finally, we diagnosed as HCV-RN. This case showed improvement of urinary abnormalities with anti-viral therapy. All cases successfully achieved remission. Thus, immunohistochemical analysis of KM55 mAb can tell us whether Gd-IgA1 is involved in the pathogenesis or not and even help us to determine therapeutic strategy.

Most of the patients with IgAN show slowly progressive clinical course. Thus, we suggest physicians to treat the comorbidity first, if its activity was severe, even if glomerular Gd-IgA1 showed positive [5]. However, the possibility of KM55-positive secondary IgAN cannot be totally excluded, because IL-6 and IL-4 accentuated galactose deficiency of IgA1 during mucosal infections [12]. Further investigations to elucidate glomerular Gd-IgA1 in case with IgAN accompanied by comorbidities are necessary.

In conclusion, present cases suggested that immunohistochemical analysis using KM55 mAb is a strong tool for differentiation of primary IgAN from secondary IgAN, and enables us to provide appropriate treatment individually.
